# Breaking tolerance in the glomerulus: complement as a driver and therapeutic target in IgA nephropathy

**DOI:** 10.3389/fmed.2026.1896069

**Published:** 2026-08-03

**Authors:** Chien-Lin Lu, Chung-Chi Yang, Chia-Chao Wu, Kuo-Cheng Lu

**Affiliations:** 1 School of Medicine, College of Medicine, Fu Jen Catholic University, New Taipei City, Taiwan; 2 Division of Nephrology, Department of Internal Medicine, Fu Jen Catholic University Hospital, Fu Jen Catholic University, New Taipei City, Taiwan; 3 Division of Cardiovascular Medicine, Taoyuan Armed Forces General Hospital, Taoyuan, Taiwan; 4 Division of Cardiovascular, Tri-Service General Hospital, National Defense Medical University, Taipei, Taiwan; 5 Graduate Institute of Microbiology and Immunology, National Defense Medical University, Taipei, Taiwan; 6 Division of Nephrology, Department of Internal Medicine, Tri-Service General Hospital, National Defense Medical University, Taipei, Taiwan; 7 Division of Nephrology, Department of Medicine, Taipei Tzu Chi Hospital, Buddhist Tzu Chi Medical Foundation, New Taipei City, Taiwan

**Keywords:** alternative pathway, complement, galactose-deficient IgA1, IgA nephropathy, lectin pathway, mucosal tolerance, Oxford MEST-C, targeted therapy

## Abstract

IgA nephropathy (IgAN) is an established autoimmune glomerular disease driven by mucosal tolerance failure, aberrant IgA1 glycosylation, and anti-glycan autoantibody formation. Mesangial deposition of galactose-deficient IgA1 (Gd-IgA1) complexes remains the central pathogenic event, though Gd-IgA1 alone is insufficient for disease, and emerging evidence implicates IgA2 deposition as an additional contributor. This review first outlines the classical, lectin, and alternative complement pathways and their predominant activation and regulatory mechanisms in IgAN, clarifying that the alternative pathway can initiate activation independently of the lectin pathway rather than acting solely as its amplifier. We then propose a compartment-based framework linking mesangial complement activation, endothelial and leukocyte responses, podocyte injury, and tubulointerstitial exposure to proteinuria, glomerulosclerosis, and fibrosis, while distinguishing complement-dependent mechanisms from complement-independent injury pathways at each site. We examine how complement biomarkers correspond to specific Oxford MEST-C lesions and discuss the current limitations of integrating these readouts with histology for risk stratification. Finally, we review pathway-selective therapies targeting mannan-binding lectin-associated serine protease (MASP)-2, factor B, C5aR, and C5, emphasizing that complement inhibition should complement rather than replace optimized supportive care and upstream IgA-axis modulation. This integrated view of autoimmune pathogenesis, complement biology, and pathway-selective therapeutics provides a foundation for precision-medicine approaches to progressive IgAN.

## 1 Introduction

IgA nephropathy (IgAN) is the most common primary glomerulonephritis worldwide and remains an important cause of progressive chronic kidney disease and kidney failure. Its prevalence, histological phenotype, and rate of progression differ across geographic and ethnic populations, with a particularly high disease burden reported in East Asian cohorts (1). Clinically, IgAN ranges from persistent microscopic hematuria to progressive proteinuria, hypertension, declining estimated glomerular filtration rate (eGFR), and kidney failure (2–4). The time course to kidney failure varies widely by cohort severity and case mix, but a substantial proportion of patients eventually require renal replacement therapy: large adult cohorts report 10-year kidney survival of roughly 78–84% and 20-year survival of roughly 64–67%, meaning that somewhere between one-sixth and one-quarter of patients progress to kidney failure within a decade, and around one-quarter to one-third do so within two decades (5, 6). Registries enriched for higher-risk patients report considerably shorter median kidney survival, around 11 to 12 years from diagnosis, compared with several decades in broader, less selected cohorts (7, 8).

This heterogeneity indicates that IgAN is not simply a passive mesangial deposition disorder. Rather, it is an autoimmune glomerular disease in which pathogenic IgA1-containing immune complexes activate local inflammatory and complement-dependent injury programs (9, 10). The accepted multi-hit model begins with increased production of galactose-deficient IgA1 (Gd-IgA1), followed by anti-glycan IgG or IgA autoantibody formation, circulating immune-complex assembly, and mesangial deposition (2, 9).

**Figure 1:**
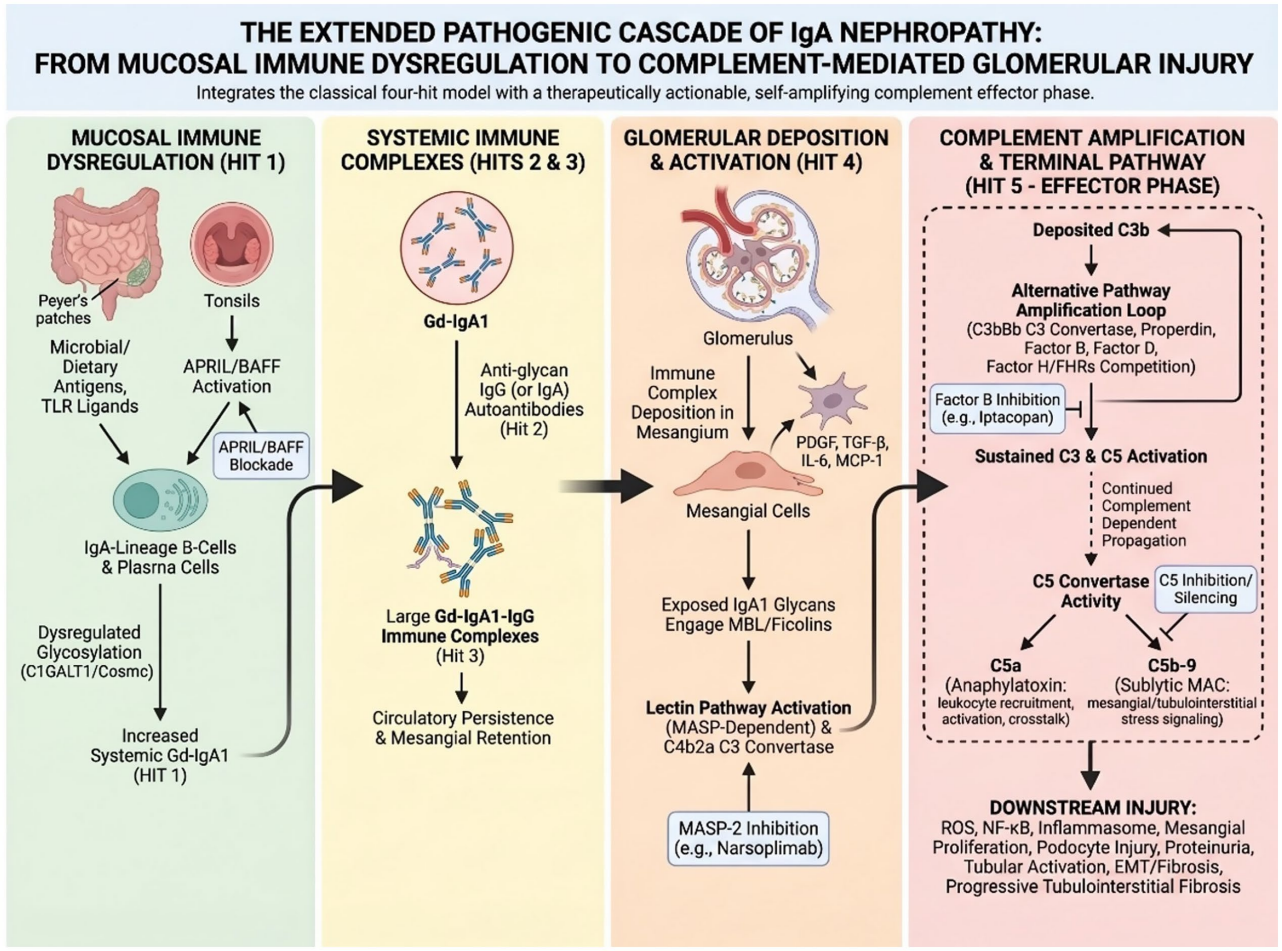
The extended pathogenic cascade of IgA nephropathy: from mucosal immune dysregulation to complement-mediated glomerular injury. The cascade integrates the classical four-hit model with a downstream complement-amplification effector phase. Mucosal antigen exposure and APRIL/BAFF signaling expand IgA-lineage B cells and drive C1GALT1/C1GALT1C1-dependent under-galactosylation of IgA1, raising systemic Gd-IgA1 (Hit 1). Anti-glycan autoantibodies bind Gd-IgA1 to form immune complexes that deposit in the mesangium, activate mesangial cells, and trigger MBL/MASP-dependent lectin pathway activation (Hits 2–4). Deposited C3b sustains factor B/D-dependent alternative pathway amplification, generating C5a and C5b-9 that drive leukocyte recruitment, mesangial/endothelial activation, and downstream injury culminating in proteinuria and tubulointerstitial fibrosis. Therapeutic targets are annotated by stage: APRIL/BAFF blockade, MASP-2 inhibition, factor B inhibition, and C5 inhibition/silencing. APRIL, a proliferation-inducing ligand; BAFF, B-cell activating factor; C1GALT1, core 1 β1,3-galactosyltransferase 1; C1GALT1C1, C1GALT1-specific chaperone 1; C5b-9, membrane attack complex; Gd-IgA1, galactose-deficient immunoglobulin A1; IgA, immunoglobulin A; IgAN, IgA nephropathy; MASP, mannan-binding lectin-associated serine protease; MBL, mannose-binding lectin.

Mesangial immune-complex deposition alone, however, does not fully explain progressive glomerular scarring or loss of kidney function. Complement activation provides the mechanistic link between deposited IgA1 complexes and active mesangial, endothelial, podocyte, and tubulointerstitial injury (9, 11–14). This review therefore frames IgAN as a mucosal autoimmune disease in which complement acts as a central effector system, integrating mucosal immune dysregulation, IgA1 glycan biology, compartment-specific complement activation, biomarker-based risk stratification, and pathway-selective therapeutics (15–17) (Figure 1).

## 2 Upstream loss of mucosal tolerance and pathogenic glycan exposure

### 2.1 Mucosal immune dysregulation and aberrant B-cell homing

The earliest immunological events in IgAN occur outside the kidney, particularly at mucosal surfaces where IgA-producing B cells are generated and programmed (18, 19). Under physiological conditions, mucosal plasma cells produce secretory IgA that neutralizes pathogens and dietary antigens at epithelial barriers with limited inflammatory activation (18, 19). Trafficking of mucosal IgA-lineage plasmablasts is regulated by chemokine and adhesion systems, including the C-C chemokine receptor 9(CCR9)- C-C motif chemokine ligand 25(CCL25) and alpha4beta7- mucosal addressin cell adhesion molecule-1 axes, which guide antibody-secreting cells toward intestinal tissue (20, 21).

In genetically susceptible individuals, mucosal infection, gut or tonsillar microbiome perturbation, and dietary antigen exposure may disrupt this compartmentalized immune program (18, 22). The resulting systemic spillover of mucosal B-cell responses increases circulating polymeric Gd-IgA1, converting a mucosal defense pathway into a systemic source of autoantigen (18, 19). This model should be interpreted as a strongly supported pathogenic framework, not as proof that every IgAN phenotype arises from identical mucosal-homing abnormalities (4, 18).

### 2.2 IgA1 O-glycosylation defects and innate pattern-recognition activation

The biochemical hallmark of pathogenic IgA1 in IgAN is aberrant O-glycosylation of the IgA1 hinge region, which generates Gd-IgA1 and exposes N-acetylgalactosamine (GalNAc)-containing neoepitopes (2, 23). Human IgA1 contains multiple hinge-region serine and threonine O-glycosylation sites; therefore, the terms “O-linked glycan chains” or “O-glycosylation sites” are preferable to “carbohydrate tracks” (23, 24). Normal IgA1 O-glycosylation requires coordinated GalNAc addition, beta1,3-galactosylation by core 1 beta1,3-galactosyltransferase 1 (C1GALT1) with its molecular chaperone C1GALT1C1/Cosmc, and subsequent sialylation (23, 25).

In IgAN, reduced or dysregulated C1GALT1/Cosmc activity and cytokine-dependent glycosyltransferase changes contribute to under-galactosylated IgA1 glycoforms that are prone to autoantibody binding (2, 25). Exposed GalNAc-rich hinge-region structures serve as autoantigenic targets for anti-glycan antibodies and may also promote innate lectin pathway recognition by mannan-binding lectin (MBL), ficolins, and mannan-binding lectin-associated serine proteases (MASPs) (9, 11, 12, 26). This dual adaptive-innate recognition process explains why systemic autoantibody formation and complement activation can occur in parallel rather than as strictly sequential events (9, 11, 27). Once deposited in the mesangium, the immune complex becomes an active enzymatic platform rather than a static structural deposit (9–11).

Gd-IgA1 is best understood as a necessary but not sufficient driver of disease: circulating and glomerular Gd-IgA1 are consistently enriched in IgAN, but unaffected first-degree relatives can also carry high serum Gd-IgA1, and clinically apparent nephropathy additionally requires anti-Gd-IgA1 autoantibody formation and nephritogenic immune-complex assembly (28, 29). Glomerular deposition of Gd-IgA1, demonstrated by KM55-based staining, is relatively specific to IgAN and IgA vasculitis with nephritis among renal diseases with IgA deposition, reinforcing its role as the central autoantigen in this disease (30). Recent evidence indicates that IgA1 is not the sole pathogenic subclass. A 2026 multicenter biopsy and humanized mouse study detected mesangial IgA2 deposition in more than 98% of IgAN biopsies, found that stronger IgA2 staining correlated with worse chronic histologic lesions and lower eGFR, and showed that humanized IgA2 mice developed greater mesangial deposition, complement C3 activation, macrophage infiltration, and tubular atrophy than IgA1 mice (31). This evidence remains less established than the core Gd-IgA1 framework, but it suggests that IgA2 may contribute an additional, partly independent injurious pathway rather than being a passive bystander subclass.

## 3 The complement system in IgA nephropathy

### 3.1 Overview of the complement activation pathways

Complement activation proceeds through three canonical routes—classical, lectin, and alternative—that converge on C3 cleavage, C5 cleavage, and assembly of the membrane attack complex (32). The classical and lectin pathways generate a shared C4b2a C3 convertase through distinct recognition mechanisms: in the classical route, C1q binds IgG- or IgM-containing immune complexes, C-reactive protein, or apoptotic surfaces and triggers sequential C1r and C1s activation (33); in the lectin route, mannose-binding lectin (MBL), ficolins, and collectins recognize carbohydrate patterns and activate MASP-2, the principal protease responsible for C4 and C2 cleavage (34). The alternative pathway differs fundamentally because it requires no dedicated recognition molecule. It is continuously seeded by spontaneous C3 hydrolysis (tickover), and factor D cleaves factor B once bound to surface C3b to form the C3bBb convertase (35).

Beyond initiation, the alternative pathway functions chiefly as an amplification loop that can dominate downstream output regardless of which pathway starts the cascade. Blocking factor D abolished more than 80% of C5a and terminal complement complex generation even when activation began through the classical pathway (36), and separate analyses estimate that the alternative pathway accounts for 80–90% of total C5b-9 production across targets (37). The boundaries between pathways are further blurred by cross-talk: MASP-3, although classified as a lectin pathway-associated protease, is the only enzyme responsible for pro-factor D activation in blood, making it indispensable for alternative pathway function (38). The three pathways are therefore best understood not as independent linear cascades but as distinct entry routes into a shared, heavily cross-regulated proteolytic network.

### 3.2 Predominant complement activation and regulatory mechanisms in IgA nephropathy

In IgAN, the alternative pathway appears to be the predominant route of glomerular complement activation, while lectin pathway involvement is confined to a smaller, more severe subset of patients (10, 39). Routine biopsy series support this hierarchy: glomerular IgA deposits are almost always accompanied by C3, whereas C1q staining is rare, arguing against a classical pathway contribution (40), and immunohistologic markers of lectin pathway activation—MBL, ficolins, MASPs, or C4d—are detected in roughly one-fourth to one-half of biopsies depending on the marker and cohort examined (40, 41). Polymeric, galactose-deficient IgA1 can activate the alternative pathway directly without lectin pathway engagement, and plasma factor Ba and C5a levels correlate with proteinuria and renal function independently of lectin markers (42). When lectin pathway markers are present, however, they track with more severe outcomes: glomerular C4d and MBL deposition are associated with heavier proteinuria, faster eGFR decline, and higher risk of kidney failure (12, 41). Therapeutic data reinforce this pathway hierarchy: factor B inhibition with iptacopan reduced proteinuria by 38.3% versus placebo at 9 months in a phase 3 trial (43), whereas MASP-2 inhibition with narsoplimab showed an early proteinuria signal in phase 2 but its phase 3 program was terminated for lack of efficacy (10).

Regulation of the alternative pathway is itself disturbed in IgAN through an imbalance between factor H and the competing factor H-related proteins factor H-related protein (FHR)-1 and FHR-5, together with loss of complement receptor 1 (11, 44). A genome-wide susceptibility locus at chromosome 1q32 captures this mechanism directly: the rs6677604-A allele, which tags deletion of CFHR3-CFHR1, is associated with higher serum factor H, lower C3a, and less mesangial C3 deposition (45). Conversely, a higher circulating FHR1-to-factor H ratio associates with progressive disease and lower eGFR, and glomerular FHR5 deposition increases while glomerular factor H decreases as IgAN advances (11, 44). Urinary complement receptor 1 (CR1) is lower in IgAN than in healthy controls while urinary membrane attack complex, factor H, and properdin are elevated, a pattern linked to podocyte-derived CR1 loss rather than filtered blood-derived CR1 (46). Experimental data support a causal role for this regulatory axis: FHR-1 increased glomerular C3 deposition and inflammatory infiltration in IgAN models, while factor H supplementation reduced IgA and C3 deposition, inflammation, and proteinuria (47, 48). This alternative pathway-centered dysregulation does not occur in full isolation from the lectin pathway: because MASP-3 is required for pro-factor D activation, activation classified as alternative pathway-driven in IgAN may still depend in part on lectin pathway machinery, and current trial data show that blocking the alternative pathway improves proteinuria without proving that lectin pathway activity was absent in the treated patients (13, 39).

## 4 Framework for the autoimmune-complement cascade

IgAN can be conceptualized as a tolerance-breakdown disorder in which complement functions as a principal effector arm of tissue injury (15, 16). The multi-hit sequence introduced in Section 2 accounts for how nephritogenic Gd-IgA1 and anti-glycan autoantibodies arise in the circulation, but it does not by itself explain why some of the resulting immune complexes, once deposited, become inflammatory and progressive rather than clinically silent; the three-tier schematic that follows addresses this latter question (49). This distinction is necessary because the multi-hit sequence alone does not account for healthy individuals with glomerular IgA deposition or asymptomatic relatives with elevated Gd-IgA1, indicating that a downstream modifier—complement regulation and activation strength—determines whether deposition becomes injurious (50, 51).

**Figure 2:**
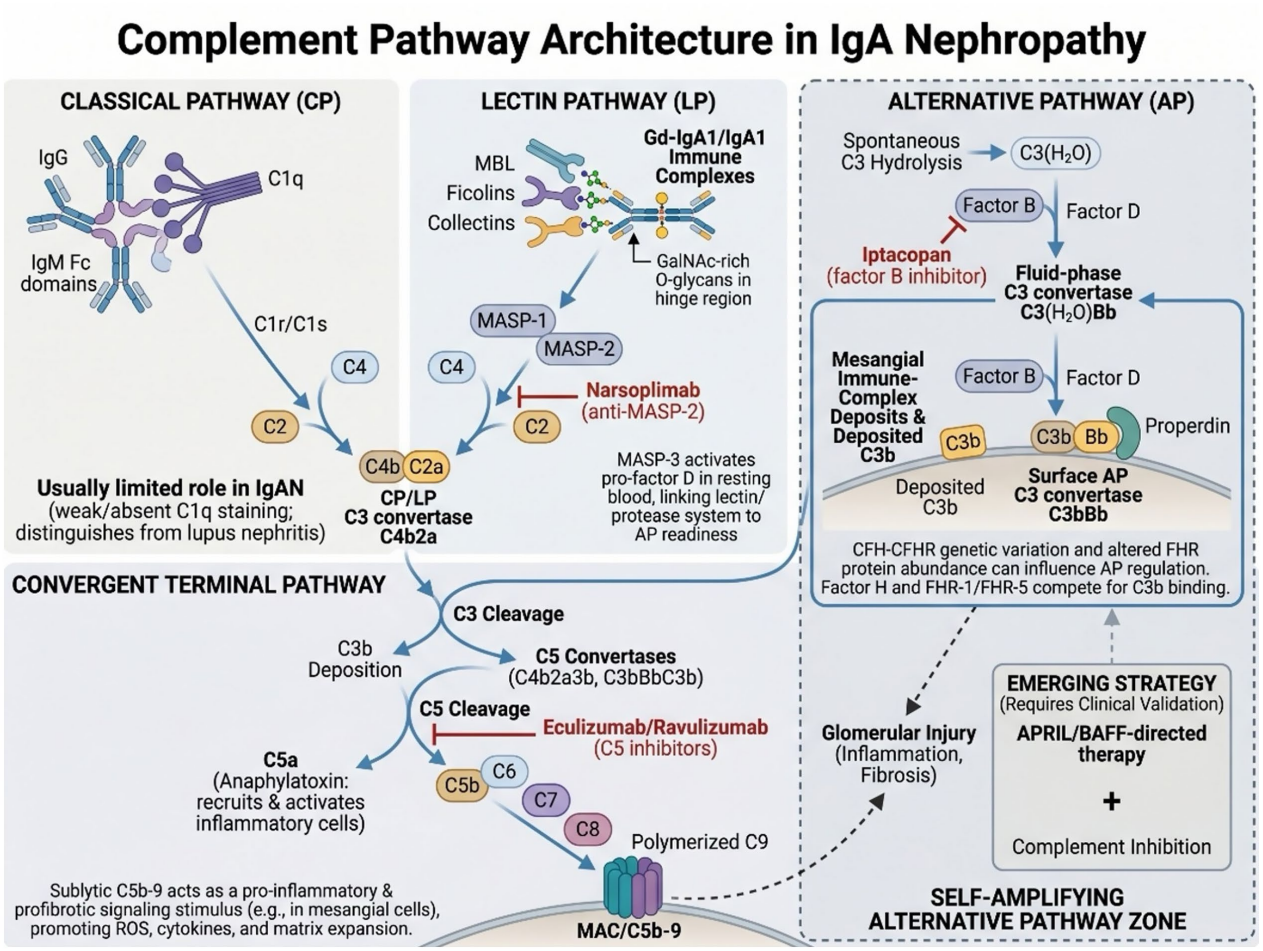
Complement pathway architecture in IgA Nephropathy. The lectin (LP) and alternative (AP) pathways are highlighted as the principal routes linking IgA1-containing immune complexes to C3b deposition, C5 activation, and C5b-9 formation; all three pathways converge at C3 cleavage, though the classical pathway has a limited role in typical IgAN given usually absent or weak C1q staining. In LP activation, GalNAc-rich O-glycans on Gd-IgA1 engage MBL, ficolins, and collectins, triggering MASP-2-mediated cleavage of C4 and C2 to form C4b2a; MASP-3 separately primes the AP via pro-factor D activation. AP amplification occurs when deposited C3b binds factor B, is cleaved by factor D, and is stabilized by properdin to form C3bBb; this loop is normally restrained by factor H, CR1, and factor I but can be enhanced by FHR-1/FHR-5 competition with factor H. Terminal pathway activation generates C5a and C5b-9, driving inflammatory-cell recruitment, mesangial activation, and profibrotic signaling. Narsoplimab targets MASP-2 at LP entry, iptacopan inhibits factor B to block AP amplification, and eculizumab/ravulizumab block C5 cleavage without preventing upstream C3b deposition. AP self-amplification remains contingent on tissue and regulatory context, and combining APRIL/BAFF-directed therapy with complement inhibition is a rational but still unvalidated hypothesis requiring prospective confirmation. AP, alternative pathway; APRIL, a proliferation-inducing ligand; BAFF, B-cell activating factor; C5b-9, membrane attack complex; CR1, complement receptor 1; FHR, factor H-related protein; GalNAc, N-acetylgalactosamine; Gd-IgA1, galactose-deficient immunoglobulin A1; IgA1, immunoglobulin A1; IgAN, IgA nephropathy; LP, lectin pathway; MASP, mannan-binding lectin-associated serine protease; MBL, mannose-binding lectin.

Tier 1, the mucosal compartment, corresponds to the upstream biology already presented in Section 2 and is summarized here only as the entry point into the framework: environmental triggers, altered B-cell programming, and C1GALT1/Cosmc-dependent overproduction of polymeric Gd-IgA1 (2, 18, 19, 22, 25). Tier 2, the circulating and mesangial compartment, depicts anti-glycan autoantibody binding, macro-immune-complex formation, and mesangial deposition (2, 9, 26). Deposited IgA1 immune complexes can induce mesangial cells to produce collectin-11, a recognition molecule that accelerates local C3 deposition rather than passively reflecting circulating immune-complex load (52), and biopsy series show that glomeruli typically display C3 and frequently C4d, but rarely C1q, favoring alternative and lectin pathway activation *in situ* over a classical pathway mechanism (39, 53). Within this glomerular activation, lectin pathway initiation and alternative pathway amplification represent two mechanistically separate steps rather than a single continuous process: MBL/MASP complexes recognize exposed glycan structures on deposited IgA1 to trigger initial C4/C2 cleavage, while properdin subsequently stabilizes the C3bBb convertase and amplifies C3 turnover independently of the initiating recognition event. Distinguishing these two steps matters because they correspond to different therapeutic targets—factor B and factor D inhibitors act on the amplification step, while MASP-2 inhibitors act on lectin pathway initiation (10, 39) (Table 1, Figure 2).

Tier 3, the compartmental injury zone, extends this activation into glomerular and tubulointerstitial compartments through a further stage of propagation rather than a direct continuation of the initial glycosylation defect, mapping sublytic C5b-9 signaling, C3a/C5a-driven endothelial activation, cytokine-mediated podocyte injury, and downstream tubulointerstitial fibrosis (9, 54–57). Mesangial deposition and the resulting complement-associated glomerular inflammation spread into tubular compartments through cell-to-cell crosstalk, abnormal protein filtration, and progressive scarring (29, 58), and this compartment-specific activity carries prognostic weight: glomerular markers including C4d, C3, C5b-9, MBL, and FHR5 show strong associations with crescentic lesions on biopsy (53, 59).

## 5 Compartment-specific pathophysiology: the mesangium

High-molecular-weight Gd-IgA1-containing immune complexes accumulate within the mesangial matrix because of their size, charge, glycan configuration, and affinity for extracellular matrix and resident mesangial-cell surfaces (2, 9). This anatomical concentration brings lectin pathway components and alternative pathway amplification proteins into close proximity with complement-responsive mesangial cells (9, 11, 12). Mesangial activation in IgAN is not solely complement-dependent: nephritogenic IgA1-IgG immune complexes engage mesangial cells directly through receptor-mediated mechanisms, including transferrin receptor (CD71) and integrin β1-linked signaling, and blocking these receptors reduces IgA-induced proliferation and cytokine production in culture (60).

Local C3 activation generates C3b and iC3b, while alternative pathway amplification promotes C5 convertase assembly and production of C5a and C5b-9 (9, 13). Much of the mechanistic detail on how sublytic C5b-9 drives mesangial injury comes from the rat Thy-1 nephritis model of mesangioproliferative glomerulonephritis rather than from IgAN tissue directly, and these findings are extrapolated to IgAN based on shared mesangial complement biology rather than direct demonstration in human IgAN specimens (61, 62). In this model, sublytic C5b-9 does not lyse mesangial cells; instead it activates TRAF6-mediated PI3K/Akt1 signaling and an ERK1/2-SOX9-Cyclin D1 pathway, both of which drive DNA synthesis and proliferation, while parallel signaling through TSP-1, transforming growth factor-β1(TGF-β1), and TIMP3 promotes fibronectin accumulation and reduced matrix turnover (62–64). Activated mesangial cells release cytokines and chemokines, including interleukin-6 (IL-6), monocyte chemoattractant protein-1 (MCP-1), TNF-alpha, and TGF-β1, thereby amplifying leukocyte recruitment and matrix remodeling (10, 54).

These processes provide a plausible route from immune-complex deposition to lesions scored under the Oxford MEST-C classification, the histological system used to grade mesangial hypercellularity (M), endocapillary hypercellularity (E), segmental sclerosis (S), tubular atrophy/interstitial fibrosis (T), and crescents (C) in IgAN biopsies. *In situ* complement activity correlates with specific lesion types rather than uniformly across all of them: C3-IgA colocalization is higher in mesangial hypercellularity (M1) and endocapillary hypercellularity (E1), and the strongest and most consistent complement association across studies is with crescents, where glomerular C5b-9, MASP1/3, MASP2, properdin, and factor B are all elevated (65). One proposed lesion-level mechanism is that complement activation proceeding to C5b-9 induces mesangial stress and production of fibronectin, TGF-*β*, and IL-6, linking immune-complex deposition to M lesions specifically (66), while associations with chronic S and T lesions are present but mechanistically less resolved, since current biopsy markers cannot reliably distinguish complement that actively drives scarring from complement deposition that simply accompanies established damage (40, 59).

## 6 Compartment-specific pathophysiology: filtration barrier and interstitium

### 6.1 Podocyte injury and endothelial activation

Although IgA deposition is concentrated in the mesangium, progressive injury extends to the glomerular filtration barrier through paracrine and complement-dependent mechanisms (10, 54). Podocytes are not the principal site of IgA immune-complex deposition; instead, experimental and clinicopathological evidence supports mesangial-derived cytokines, particularly TNF-alpha and TGF-*β*, as important mediators of podocyte stress and injury. Podocyte pathology is therefore best described by reduced nephrin and podocin expression, actin cytoskeletal remodeling, foot-process effacement, and detachment, rather than by direct IgA uptake alone (10, 54, 67).

Endothelial injury in IgAN is not exclusively complement-driven. Gd-IgA1-containing immune complexes, rather than free Gd-IgA1, directly activate glomerular endothelial cells in mice and in cultured human glomerular endothelial cells, producing glycocalyx loss, increased adhesion-molecule and cytokine expression, and greater permeability that favors immune-complex entry into the mesangium (68). A separate mesangial-endothelial crosstalk pathway also operates independently of direct complement attack on endothelium: circulating IgA1 from patients increases IL-6 and CXCL1 release from mesangial cells, and the resulting conditioned medium raises endothelial injury markers including von Willebrand factor, soluble VCAM-1, and HB-EGF (69). Complement nonetheless remains a major contributor at this site: C3a and C5a fragments activate endothelial and leukocyte responses through local C3a receptor (C3aR) and C5a receptor (C5aR) signaling, promoting endothelial activation, leukocyte adhesion, and endocapillary hypercellularity (56, 57, 70), and C3 co-deposition is detected in more than 90% of IgAN biopsies, with its absence associated with a more benign clinical course (29). These complement-dependent and complement-independent mechanisms together help explain how a predominantly mesangial immunodeposit can produce filtration-barrier dysfunction and proteinuria.

**Table 1:** Structural components of complement activation in IgAN.

Component	Gene/shorthand	Main localization	Mechanistic role
Galactose-deficient IgA1	C1GALT1/C1GALT1C1 context; Gd-IgA1	Circulation and mesangial immune complexes	Primary autoantigenic substrate with exposed GalNAc-rich hinge-region glycans (2, 23, 29).
Mannose-binding lectin	MBL2; MBL	Circulating complexes and mesangial deposits	Pattern-recognition molecule implicated in lectin pathway activation in IgAN (11, 12).
MASP-2	MASP2	Lectin pathway complexes	Cleaves C4/C2 after lectin pathway recognition and is the therapeutic target of narsoplimab (12, 101).
Properdin	CFP	Alternative pathway convertases	Stabilizes the alternative pathway C3 convertase (C3bBb) and amplifies loop activity (9, 16).
C3a	C3 cleavage product	Glomerular and tubular inflammatory milieu	An anaphylatoxin that supports leukocyte, endothelial, and tubular activation loops (56, 57, 70).
C5a	C5 cleavage product	Endothelium and inflammatory infiltrates	Potent anaphylatoxin that supports leukocyte recruitment and endocapillary inflammation (13, 56, 70).
C5b-9 / MAC	C5b-C9	Mesangial cell membranes and urine	Sublytic insertion promotes mesangial activation; urinary excretion tracks structural damage (16, 55, 57).

Key structural and regulatory components involved in complement activation in IgA nephropathy, including their genetic shorthand, localization, and mechanistic roles in lectin, alternative, and terminal pathway signaling. Gd-IgA1, galactose-deficient immunoglobulin A1; GalNAc, N-acetylgalactosamine; MBL, mannose-binding lectin; MASP-2, mannan-binding lectin-associated serine protease-2; MAC, membrane attack complex.

**Table 2:** Complement biomarkers and cautious alignment with Oxford MEST-C lesions.

Biomarker	Matrix	Main source/pathway	Validated interpretation
Soluble C5b-9 (sC5b-9)	Serum/plasma	Terminal pathway activation and systemic MAC assembly	Reflects terminal complement activation and may identify more active inflammatory phenotypes; interpretation should be integrated with clinical and biopsy data (9, 55, 57).
Urinary C5b-9	Spot or 24-h urine	Filtered or locally generated terminal complement complexes	Associated with renal injury and progression; tracks proteinuria and tubulointerstitial damage, but absolute correlation wording should be avoided (46, 55, 57).
Glomerular C4d	Kidney biopsy tissue	Lectin pathway activation marker	C4d deposition is associated with adverse kidney outcomes and supports lectin pathway involvement in IgAN (59, 78, 83).
FHR-5 / Factor H-related proteins	Serum and tissue context	Alternative pathway regulation and Factor H competition	FHR-5 has been associated with Oxford M and E lesions, overall MEST severity, and histologic injury (102, 103).
C3a/C3aR and C5a/C5aR	Plasma, tissue, urine context	Anaphylatoxin generation downstream of C3/C5 convertases	Receptor deposition or activation has been linked to advanced IgAN progression and endothelial/leukocyte activation, particularly in endocapillary inflammation (56, 57, 70).

Complement biomarkers in IgA nephropathy and their cautious alignment with Oxford MEST-C histological lesions, emphasizing tissue, serum, and urinary readouts relevant to disease activity and progression. FHR-5, factor H-related protein 5; IgAN, immunoglobulin A nephropathy; MEST-C, Mesangial hypercellularity, Endocapillary hypercellularity, Segmental glomerulosclerosis, Tubular atrophy/interstitial fibrosis, and Crescents; sC5b-9, soluble C5b-9.

**Table 3:** Targeted complement-inhibitor pipeline in IgAN.

Agent	Target/class	Mechanism	Validated identifiers	Status/interpretation
Iptacopan (LNP023)	Factor B; oral small-molecule inhibitor	Blocks alternative pathway C3/C5 convertase amplification loops.	Phase 3 APPLAUSE-IgAN; ClinicalTrials.gov: NCT04578834 (43).	Phase 3 data demonstrate significant proteinuria reduction; regulatory statements should distinguish accelerated approval based on proteinuria from confirmatory kidney-function outcomes.
Narsoplimab (OMS721)	MASP-2; monoclonal antibody	Inhibits lectin pathway initiation by blocking MASP-2 protease activity.	Phase 2 data; NCT02682407; phase 3 program NCT03608033 (101).	Phase 2 data provided early clinical proof-of-concept, but the phase 3 ARTEMIS-IGAN trial was discontinued in 2023 after an interim analysis showed no statistically significant proteinuria benefit over placebo, despite an acceptable safety profile (10).
Ravulizumab	C5; long-acting monoclonal antibody	Blocks C5 cleavage into C5a and C5b, reducing terminal pathway activation and MAC assembly.	Phase 2 data; phase 3 registry NCT06291376 (92).	Phase 2 trial supports terminal blockade in IgAN.
Cemdisiran (ALN-CC5)	C5 mRNA; GalNAc-conjugated siRNA	Suppresses hepatic C5 synthesis and reduces circulating C5 availability.	Phase 2 RCT; ClinicalTrials.gov: NCT03841448 (104).	Completed phase 2 randomized trial evaluated proteinuria and safety in adults with IgAN.
OMS906	MASP-3; monoclonal antibody	Targets MASP-3 to modulate alternative pathway priming and activation.	Early clinical; ClinicalTrials.gov: NCT05646199 (16).	Active early-stage clinical development; an upstream approach to alternative pathway modulation.

Emerging complement-targeted therapies in IgA nephropathy, summarizing therapeutic targets, mechanisms of action, clinical-trial identifiers, and current translational interpretation. GalNAc, N-acetylgalactosamine; IgAN, immunoglobulin A nephropathy; MAC, membrane attack complex; MASP-2, mannan-binding lectin-associated serine protease-2; MASP-3, mannan-binding lectin-associated serine protease-3; RCT, randomized controlled trial; siRNA, small interfering RNA.

### 6.2 Tubulointerstitial injury

Filtered complement fragments, proteinuria, and downstream inflammatory mediators expose proximal tubular epithelial cells to chronic stress in progressive IgAN (57, 71). Tubular injury in this setting is not attributable to complement fragments alone: proteinuria itself injures tubular epithelial cells through protein overload, oxidative and endoplasmic reticulum stress, and maladaptive remodeling, and several non-complement urinary proteins track this injury independently. Urinary DKK3 independently predicts interstitial fibrosis and greater eGFR decline beyond the effect of proteinuria alone in biopsied proteinuric kidney disease, while MCP-1 and KIM-1 correlate with cortical interstitial inflammation and proximal tubular injury, respectively (72). Injured tubular epithelial cells undergo activation, dedifferentiation, and inflammatory signaling (71, 73). Lineage-tracing studies indicate that most kidney myofibroblasts arise from resident interstitial fibroblasts, pericytes, and related mesenchymal stromal populations rather than from complete tubular epithelial-mesenchymal transition (74, 75). The injured tubular epithelial cells undergo partial epithelial-mesenchymal transition or fibrogenic reprogramming while secreting profibrotic cytokines such as TGF-β1 (71, 73, 76).

These paracrine signals activate interstitial fibroblasts and pericytes, leading to collagen deposition, capillary rarefaction, tubular atrophy, and progressive interstitial fibrosis (71, 75, 77). Because tubulointerstitial scarring strongly predicts long-term renal survival, urinary complement biomarkers, interpreted alongside non-complement tubular markers such as uDKK3 and KIM-1, may together provide more complete information on active compartment-specific damage than complement markers alone (55, 57, 72).

## 7 Translational context: biomarkers and Oxford MEST-C alignment

The Oxford MEST-C classification provides the international histological framework for IgAN risk stratification by scoring mesangial hypercellularity, endocapillary hypercellularity, segmental sclerosis, tubular atrophy/interstitial fibrosis, and cellular or fibrocellular crescents (4, 59, 78). The score is useful but imperfect, and its components do not carry equal weight. T is the most reproducible and most consistently prognostic lesion across validation cohorts, whereas interobserver agreement for M, E, and C is frequently poor in multicenter practice: in the VALIGA cohort, agreement between local and central pathologists was poor for M, E, and C and only moderate for S and T, with local pathologists scoring more M, E, and C lesions and fewer S lesions than central reviewers (79, 80). Disagreement is most frequent when only a small proportion of glomeruli show the lesion in question, pointing to threshold and sampling effects rather than random scoring error alone (79). The score also has conceptual gaps: crescents were incorporated without formal reproducibility testing, and tubulointerstitial inflammation, which appears clinically relevant, is not captured at all (80, 81). These limitations mean complement biomarkers cannot simply substitute for histology, but they raise the question of whether complement readouts might compensate for some of the reproducibility gaps that limit MEST-C on its own.

Complement biomarkers should not be interpreted as nonspecific inflammatory markers; distinct tissue, serum, and urinary markers track different compartments and activation loops, and several now align with specific MEST-C lesions rather than with disease activity in general (Table 2) (9, 57, 59, 82). C3-IgA colocalization is higher in mesangial hypercellularity (M1) and endocapillary hypercellularity (E1), and the most consistent complement association across studies is with crescents: a 2023 systematic review found every examined complement factor associated with C1-2, and a separate biopsy study linked crescents to higher glomerular C5b-9, MASP1/3, MASP2, properdin, and factor B (65). Tissue C4d and lectin pathway components align most closely with complement-active mesangial and crescentic injury patterns (78, 83). Associations with chronic S and T lesions are also reported, but they are mechanistically less settled, since most biopsy markers cannot distinguish complement that actively drives scarring from complement deposition that simply accompanies already-established damage (40, 59). Urinary C5b-9 and related terminal pathway fragments may reflect filtration-barrier disruption, proteinuria, and tubulointerstitial involvement, although absolute threshold-based claims should be avoided until prospectively validated (55, 57).

Integrating Oxford MEST-C scoring with serum, urinary, and tissue complement readouts could in principle refine risk stratification and support selection of patients for pathway-selective complement-targeted therapies, but this remains an unrealized goal rather than an established practice (15, 16, 59, 84). The clearest barrier is that routine biopsy staining for complement deposits cannot distinguish past from ongoing activation; newer *in situ* convertase assays address this by directly measuring active C3 and C5 convertases in tissue, and one such study found that convertase signal correlated with mesangial C5b-9 and was more intense in E1 and S1 lesions, suggesting complement activity at biopsy is not only a static deposit but an ongoing process (85). This kind of dynamic assay remains rare, and most evidence linking complement to MEST-C is still cross-sectional immunostaining rather than functional or longitudinal data (86). Reviews consistently describe complement as a rational treatment target in IgAN, yet they also acknowledge that the precise mechanisms linking specific pathways to specific lesions, and the criteria for selecting patients by lesion type rather than by global MEST-C score, remain incompletely defined (39, 51). Closing this gap will likely require combining reproducible histological components, such as T, with pathway-specific complement assays validated against prospective renal outcomes rather than against MEST-C scores alone.

## 8 Complement-targeted immunotherapies

The therapeutic landscape of IgAN is shifting from broad, non-specific immunosuppression toward mechanism-based strategies that modify upstream mucosal autoimmunity, pathogenic immune-complex formation, and complement-mediated effector injury (15, 16, 87). Complement inhibitors can be organized into proximal pathway inhibitors, which block C3 convertase formation or AP amplification, and terminal pathway inhibitors, which prevent C5a generation or C5b-9 assembly (Table 3) (11, 16, 88). Factor B inhibition with iptacopan has the most mature evidence in this class: in the phase 3 APPLAUSE-IgAN trial, 24-h urine protein-to-creatinine ratio fell by 38.3% relative to placebo at 9 months in patients already on optimized supportive care, with a comparable 38.0% reduction observed in an East Asian subgroup analysis (43, 89). An earlier phase 2 trial established dose dependence, with a 23% urine protein creatinine ratio (UPCR) reduction at the 200 mg twice-daily dose by 3 months and further decline by 6 months, alongside sustained reductions in plasma Bb and urinary C5b-9 confirming target engagement (90). Complement inhibitors as a drug class reduce proteinuria by approximately 35% across randomized trials in meta-analysis, though confirmatory data on long-term eGFR-slope benefit remain pending (91).

Terminal complement inhibition at C5 has also been evaluated in IgA nephropathy. In a phase 2, randomized, double-blind, placebo-controlled trial (NCT04564339), 66 adults with biopsy-confirmed IgAN, proteinuria ≥1 g/day, and eGFR ≥30 mL/min/1.73 m^2^ on stable maximally tolerated renin-angiotensin blockade were randomized 2:1 to the long-acting anti-C5 monoclonal antibody ravulizumab or placebo for 26 weeks. A statistically significant 30.1% (90% CI, 13.7–43.5%) relative reduction in proteinuria was observed with ravulizumab versus placebo at approximately 6 months, alongside a favorable safety profile (92). These findings provide clinical proof-of-concept that terminal pathway blockade at C5 can produce a rapid and clinically meaningful reduction in proteinuria in IgAN, complementing the evidence for upstream alternative pathway inhibition with iptacopan, and have motivated the ongoing phase 3 program (NCT06291376) that will assess whether this early biomarker signal translates into preservation of long-term kidney function.

The risk of infection from encapsulated bacteria is not unique to terminal C5 blockade. Proximal alternative pathway inhibitors such as factor B antagonists carry a comparable theoretical risk, because complement blockade at any point upstream of the membrane attack complex can impair host defense against *Neisseria meningitidis* and other encapsulated organisms, and reviews of complement inhibition in IgAN identify vaccination against encapsulated bacteria as a likely requirement across this drug class rather than a C5-specific precaution (93, 94). This recommendation remains precautionary rather than a response to an observed excess of infections in current trials: phase 3 iptacopan reported adverse-event rates similar to placebo with no increased infection signal, and the phase 2 trial recorded no bacterial infections or treatment-related serious adverse events (43, 90). Selective alternative pathway blockade has a mechanistic rationale for a lower infection burden than broader inhibition, since it leaves classical and lectin pathway activation intact, but the supporting safety data remain short-term and concentrated in a single agent rather than established across the class (94).

Beyond C3 and C5-directed agents, two additional complement targets have produced clinical evidence in IgAN. Antisense oligonucleotide suppression of hepatic factor B synthesis reduced systemic factor B levels by up to 69% with repeated dosing in early-phase studies, and an open-label phase 2 trial reported proteinuria reduction of approximately 43–45% by week 29, although this evidence remains uncontrolled and single-arm rather than placebo-confirmed (95). At the receptor level, the C5a receptor antagonist avacopan has been evaluated in a small open-label pilot study of seven patients with biopsy-proven IgAN and persistent proteinuria despite maximally tolerated renin-angiotensin-aldosterone system blockade: six of seven patients showed numerical UPCR improvement and three reached approximately 50% reduction by week 12, with urinary MCP-1-to-creatinine ratio falling by about 30% by week 8 (96). A 2023 review called for longer-term controlled study before efficacy in IgAN can be considered established (39). Furthermore, interpretation of this comparator has since been complicated by the June 29, 2026 retraction of the pivotal ADVOCATE phase 3 trial of avacopan in ANCA-associated vasculitis by the New England Journal of Medicine, following an FDA investigation that identified undisclosed post-database-lock primary endpoint readjudications inconsistent with proper research conduct (97). In parallel, the U. S. FDA has proposed withdrawal of avacopan’s approval and the European Medicines Agency’s Committee for Medicinal Products for Human Use (CHMP) has recommended revocation of its EU marketing authorization, concluding that the ADVOCATE study was conducted in breach of Good Clinical Practice principles and that the drug’s benefit–risk balance in AAV can no longer be considered established [CDER, EMA]. These developments remove the AAV benchmark previously used to contextualize C5aR1 blockade in IgAN, and the small IgAN pilot data should therefore be interpreted purely on their own limited merits pending adequately powered controlled study. Preclinical work supports the mechanistic rationale for C5aR1 blockade, since C3aR or C5aR knockout reduced mesangial IgA deposition in animal models (98). Antimicrobial prophylaxis and vaccination timing should be individualized according to local guidance, drug-specific risk-mitigation programs, resistance patterns, and patient-specific infection risk rather than described as a fixed-duration universal requirement (99, 100).

## 9 Limitations

Although accumulating evidence highlights complement activation in IgAN, several limitations persist: current data rely primarily on observational and translational studies rather than established causal links, and significant biological heterogeneity exists, as lectin pathway markers are inconsistently expressed and alternative pathway amplification varies by genetic and inflammatory context. Much of the mechanistic detail connecting complement activation to mesangial and endothelial injury discussed in this review is extrapolated from animal models, particularly the rat Thy-1 nephritis model of mesangioproliferative glomerulonephritis, rather than demonstrated directly in human IgAN tissue, so the applicability of these signaling pathways to patients still requires direct confirmation. Consequently, complement biomarkers must be interpreted as indicators of specific complement-active phenotypes rather than universal disease markers. Furthermore, the clinical utility of these serum, tissue, and urinary biomarkers is restricted by unstandardized assay platforms, variable sampling times, and a lack of validated therapeutic thresholds. In addition, most evidence linking complement biomarkers to specific Oxford MEST-C lesions remains cross-sectional immunostaining rather than longitudinal or functional data, so current findings cannot reliably distinguish complement activity that is actively driving injury from complement deposition that merely accompanies established damage. While emerging complement-targeted interventions targeting MASP-2, factor B, and C5 show promise, their long-term efficacy, safety profiles, and patient selection criteria require prospective clinical trial validation. Finally, the proposed compartment-based pathogenic model integrating mucosal dysregulation, immune-complex deposition, and renal injury remains conceptual and must be iteratively refined as novel mechanistic and clinical evidence emerges.

## 10 Conclusion

IgAN is an autoimmune glomerular disease in which mucosal immune dysregulation and aberrant IgA1 glycosylation generate nephritogenic immune complexes that deposit in the mesangium, with Gd-IgA1 as the central but not sufficient driver and IgA2 deposition emerging as an additional contributor. Complement activation then converts this deposition into compartment-specific glomerular and tubulointerstitial injury through lectin and alternative pathway activation, the latter capable of initiating independently rather than only amplifying, anaphylatoxin generation, and terminal C5b-9 signaling. Integrating complement biomarkers with Oxford MEST-C histology may eventually refine risk stratification and guide pathway-selective therapy, though this remains an unrealized goal rather than current practice. As complement-targeted agents mature, the priority is to deploy them as a precision layer added to optimized supportive care and upstream IgA-axis modulation, not as a replacement, with future trials needed to define which patients benefit most and how to balance renal protection against infectious risk.

## Glossary

ALN-CC5: Cemdisiran
C1GALT1: Core 1 beta1,3-galactosyltransferase 1
C1GALT1C1: C1GALT1-specific chaperone 1
C3aR: C3a receptor
C5aR: C5a receptor
C5b-9: Terminal complement complex
CCL25: C-C motif chemokine ligand 25
CCR9: C-C chemokine receptor 9
CR1: Complement receptor 1
eGFR: Estimated glomerular filtration rate
FHR: Factor H-related protein
GalNAc: N-acetylgalactosamine
Gd-IgA1: Galactose-deficient immunoglobulin A1
IgA, Immunoglobulin A
IgA1: Immunoglobulin A1
IgAN: IgA nephropathy
IL-6: Interleukin-6
MASP, Mannan-binding lectin-associated serine protease
MBL, Mannose-binding lectin
MCP-1: Monocyte chemoattractant protein-1
TGF-β1: Transforming growth factor-β1
